# Strengthening and enhancing national antiretroviral drug resistance surveillance in Zimbabwe—A country that has reached UNAIDS 95-95-95 amongst adults

**DOI:** 10.3389/fpubh.2024.1346027

**Published:** 2024-02-14

**Authors:** Tafadzwa Dzinamarira, Enos Moyo, Brian Moyo, Grant Murewanhema, Diego Cuadros, Vinie Kouamou, Amon Mpofu, Godfrey Musuka

**Affiliations:** ^1^School of Health Systems and Public Health, University of Pretoria, Pretoria, South Africa; ^2^International Center for AIDS Care and Treatment Programs, Columbia University, Lusaka, Zambia; ^3^Department of Public Health Medicine, University of KwaZulu Natal, Durban, South Africa; ^4^Ministry of Health and Child Care, Harare, Zimbabwe; ^5^Faculty of Medicine and Health Sciences, University of Zimbabwe, Harare, Zimbabwe; ^6^University of Cincinnati, Cincinnati, OH, United States; ^7^National AIDS Council of Zimbabwe, Harare, Zimbabwe; ^8^International Initiative for Impact Evaluation, Harare, Zimbabwe

**Keywords:** HIVDR, surveillance, Zimbabwe, antiretroviral therapy, public health

## Introduction

AIDS claimed a life every minute in 2022. Millions of people still miss out on treatment, including 43% of children living with HIV (1). It is inspiring to note that Botswana, Eswatini, Rwanda, the United Republic of Tanzania, and Zimbabwe have already achieved the 95-95-95 targets amongst adults. At least 16 other countries (including eight in sub-Saharan Africa) are close to doing so (1). Achieving epidemic control is now within reach. Financing the response to HIV infection may increasingly become more challenging in resource-poor countries with a high disease burden that will have attained 95-95-95 amongst adults as donor resources are directed to other poor countries with less robust HIV response programs (2).

Zimbabwe, a low-income country in southern Africa with a population of more than 15 million, has been hard hit by HIV infection and had a peak prevalence of 26% in adults in the late 1990s (3). In 2021, 96% of adults knew their status, 96% of adults living with HIV were on antiretroviral treatment (ART), and 93% of adults on treatment had suppressed viral load. To sustain these gains as it approaches ending HIV, there is a need for the country to strengthen HIV prevention using both long-established approaches such as condom programming, Voluntary medical male circumcision (VMMC), and early infant diagnosis as well as more recent innovations such as Pre-exposure prophylaxis (PrEP).

However, of concern was that children in Zimbabwe were still lagging in achieving the 95-95-95 targets due to ingrained inequalities and a lack of innovative case finding. For example, in 2021, 73% of children living with HIV in Zimbabwe knew their HIV status, 73% of children living with HIV were on treatment, and only 58% had suppressed viral load (4). Going forward Zimbabwe will need to redouble efforts to ensure that children are not left behind in the HIV response.

For the country to be less dependent on donor funding for its HIV response, in 1999, the Zimbabwean government introduced a National AIDS Trust Fund or “AIDS Levy (5).” Through a 3% tax on formal sector income and business profits (which excluded the mining sector until 2015), the AIDS Levy has raised well over US$100 million for the national response to HIV and AIDS, including US$38.6 million in 2014 alone (6). Going forward Zimbabwe will need to depend on domestic resources such as those generated by the AIDS Levy to sustain its HIV programs.

To sustain the gains made over the years to end the HIV epidemic, efforts should be reinforced on prevention through the use of pre-exposure prophylaxis (PrEP), on monitoring viral load among individuals on ART, and finally on surveillance of HIV drug resistance (HIVDR) including pretreatment resistance (among patient in initiating ART) and acquired HIVDR (among patients failing on their current regimens). HIVDR refers to the ability of the virus to mutate and reproduce in the presence of antiretroviral drugs, rendering these drugs less effective or even ineffective. This resistance develops when HIV replicates in the presence of antiretroviral drugs, but due to incomplete viral suppression, selective pressure allows resistant strains to thrive (7). HIVDR is a significant concern because it can lead to treatment failure, limiting options for effective HIV management. The emergence of HIVDR is often associated with poor adherence to ART, suboptimal drug regimens, and transmission of drug-resistant strains (8). It poses a challenge to the global goal of controlling the HIV epidemic, as it can compromise the efficacy of first-line ART regimens, necessitating the use of more complex and costly second-line or third-line therapies.

The prevalence of HIVDR is a growing concern globally, particularly in regions with widespread access to antiretroviral therapy (ART). Africa has witnessed a significant increase in ART availability, accompanied by a rise in HIVDR cases (9–11). This is particularly concerning in newly infected individuals and those starting ART in sub-Saharan Africa, as it impacts the effectiveness of first-line treatment regimens and threatens the overall success of HIV control programs. Zimbabwe mirrors the challenges faced across Africa, despite efforts to control HIV spread and improve treatment outcomes (12–14). Studies show some success in managing resistance, with primary drug resistance prevalence in HIV-infected pregnant women below the WHO threshold. However, the overall drug-resistance prevalence in Zimbabwe is around 5.6% (15), including a high prevalence of drug-resistant mutations in HIV-infected infants, raising concerns for future treatment strategies (16). Moderate levels of pre-treatment HIVDR have also been observed, affecting the ability to achieve viral suppression in patients initiating ART (17). These statistics highlight the ongoing challenge of managing HIVDR in Zimbabwe, emphasizing the need for robust surveillance, effective treatment strategies, and adherence to ART to mitigate the spread and impact of drug-resistant HIV strains.

HIVDR can be transmitted or acquired through various means, including previous antiretroviral exposure (18). Multiple factors contribute to HIVDR emergence, including viral, drug-related, programmatic, and patient-related factors. Increased pretreatment HIVDR prevalence is linked to ART failure, potentially leading to further resistance (19, 20). Previous Zimbabwe studies show high pretreatment and acquired HIVDR against non-nucleoside reverse transcriptase inhibitors. Dolutegravir is the preferred first-line regimen but enhanced HIVDR surveillance is crucial to preserve its efficacy (12, 21, 22). In this article, we discuss the need, benefits, and strategies for strengthening and enhancing HIVDR surveillance in Zimbabwe.

## The need for strengthening and enhancing HIVDR surveillance

The WHO recommends the development of a national action plan on HIVDR, annual monitoring of Early Warning Indicators (EWIs) and routine HIVDR surveys (including, pre-treatment and acquired HIVDR surveys and HIVDR among PrEP users diagnosed with HIV every 3 to 5 years) (21). Limited funding has hindered these activities in Zimbabwe. Funding for HIV programs is anticipated to continue contracting as the country approaches epidemic control (2).

As ART and PrEP expand, HIVDR emergence is likely, to jeopardize HIV/AIDS eradication efforts. Dolutegravir (DTG), though known for its high genetic resistance barrier, has shown resistance elsewhere, particularly among ART-experienced individuals (23). While a recent Zimbabwe study found no DTG resistance among ART-naïve individuals (24), surveillance and monitoring of HIVDR among DTG users is crucial due to its widespread use and potential future treatment limitations (25, 26). HIVDR survey results inform program performance, guide second-line therapy selection, and prevent unnecessary therapy switching.

## The benefits of strengthening and enhancing HIVDR surveillance

Strengthening and enhancing HIVDR surveillance has several benefits. Particularly in low-to-middle-income countries (LMICs) that do not conduct resistance testing before starting ART, knowledge of pretreatment HIVDR at the population level can help countries choose effective first-line ART, drugs for PrEP, and post-exposure prophylaxis (18).

When the anchor drugs are susceptible, HIVDR surveillance for individual patient monitoring may reduce the number of unnecessary therapy switches. It should be highlighted that the implementation of HIVDR surveillance at the patient level in LMICs like Zimbabwe only adds value when viral load testing is available on a large scale, resources, and laboratory capacity are available, and the country has access to clinical experts and virologists who can interpret the resistance results (20). When routine HIVDR is not possible because of several factors, including technological and financial limitations, as is the case in Zimbabwe, it is especially recommended that regular HIVDR surveys be conducted. There will be certain patient groups that require monitoring in these circumstances. Such groups include adults initiating or re-initiating first-line ART, infants newly diagnosed with HIV, anyone with virological failure, and HIV-diagnosed PrEP recipients (20). It is also important to note that, without surveillance, patients may be maintained on failing regimes, which leads to the accumulation of resistance mutations that jeopardize the effectiveness of ensuing regimens (8). HIVDR therefore improves treatment outcomes as patients are switched to effective regimens on time when their current regimens are failing, resulting in a reduction in the accumulation of resistance mutations that may compromise future regimens. This will also prolong the useful life of first-line regimens, thereby reducing the costs of ART programs (8).

The best salvage therapy can be informed by genotypic resistance testing (20). Genotypic resistance testing helps in predicting antiretroviral drug susceptibility and subsequent effective treatment for patients in the future (27). Additionally, surveillance can be used to identify factors that are possibly associated with the emergence of HIVDR. Such information is crucial in designing and implementing HIVDR prevention strategies (28). With DTG being given as the first-line ART, there are limited ART options for third-line regimens in LMICs such as Zimbabwe. HIVDR testing and surveillance are necessary for patients who are not responding to protease inhibitor-based ART (20). In addition to benefiting patients, HIVDR surveillance offers vital data that are needed to evaluate how well ART programs are doing at achieving viral suppression targets (20).

## Strategies for strengthening and enhancing HIVDR surveillance

Effective surveillance is crucial for identifying emerging resistance patterns, informing treatment guidelines, and ensuring the continued efficacy of antiretroviral therapies. Regular monitoring of patients on ART for signs of drug resistance, including both genotypic and phenotypic resistance testing, is necessary. Additionally, establishing robust systems for collecting and analyzing data on HIVDR is vital. This process involves gathering data from various sources such as hospitals, clinics, and laboratories, and utilizing digital health records and databases can streamline this process and facilitate more effective data analysis.

Research and development in new methods of surveillance for HIVDR are also key elements for enhancing the effectiveness of HIV treatment programs and controlling the spread of HIVDR. This field is continuously evolving, with areas of focus including molecular techniques for drug resistance testing, point-of-care testing (POCT), bioinformatics and data analysis tools, surveillance of HIVDR, integration with digital health platforms, patient monitoring technologies, and epidemiological studies (7, 29). Spatial analysis, which involves examining the locations, attributes, and relationships of features in geographic space, can provide critical insights into the patterns, causes, and effects of health phenomena like HIVDR (30). This includes identifying hotspots of resistance, understanding transmission dynamics, and informing resource allocation and planning. By focusing on these advanced techniques and tools, healthcare systems can significantly enhance their ability to monitor and respond to HIVDR, ultimately improving treatment outcomes and controlling the spread of HIV (31, 32).

Some strategies to strengthen and enhance HIVDR surveillance include increasing viral load monitoring among patients on ART, training healthcare workers to identify patients who require HIVDR testing, ensuring that surveys of HIVDR are conducted routinely, accreditation of laboratories that perform HIVDR testing, and the monitoring program quality indicators, also known as EWIs (33). Although Zimbabwe has already achieved the UNAIDS 95-95-95 targets in adults and not yet in children, strategies should be put in place to conduct surveys of HIVDR routinely, to monitor the emergence and transmission of HIVDR, measure the degree to which program practices minimize the emergence and transmission of HIVDR, and provide data to support the selection of optimal ART regimens. These surveys should be nationally representative (33).

To evaluate clinic-level data and pinpoint programmatic deficiencies, Zimbabwe should also monitor EWIs. EWIs can be tracked at representative sites or across all ART sites in the country (34). EWIs are clinic, patient, and program factors that serve as a sentinel for HIVDR emergence (35). Early detection of these variables can assist HCWs in implementing the proper corrective measures at the clinic level. It can also serve as a warning to program managers and policymakers to take the necessary precautions to stop HIVDR development on a national scale (36). The WHO advises annual HIVDR EWI surveillance at all ART clinics or a representative sample of all healthcare institutions in the country (34). However, in LMICs like Zimbabwe, EWIs can be monitored every 2 or 3 years due to a lack of resources. Facilities that exhibit poor performance for any EWI should be identified for programmatic follow-up (37). Additionally, EWIs must be incorporated into regular national monitoring and evaluation tasks related to ART programs (33). The integration of HIVDR surveillance into national HIV/AIDS control programs ensures that surveillance is a regular part of HIV care and treatment (38).

## Conclusion

Effective HIVDR management requires comprehensive surveillance, encompassing patient monitoring, data analysis, program integration, and healthcare worker training. Advances in drug resistance testing, point-of-care testing, and bioinformatics tools enhance surveillance and treatment strategies. EWIs and spatial analysis identify and address factors contributing to HIVDR, ensuring effective and adaptable treatment.

In Zimbabwe, the situation of HIVDR mirrors the complexities faced globally, with unique regional challenges. Zimbabwe's response to HIVDR involves not only addressing these factors but also tailoring strategies to its specific epidemiological and socio-economic context. This includes enhancing local capacity for drug resistance testing, improving patient adherence through community engagement and education, and ensuring the availability of effective first and second-line treatment options.

## Author contributions

TD: Conceptualization, Writing—original draft. EM: Writing—original draft. BM: Writing—review & editing. GM: Writing—original draft. DC: Writing—review & editing. VK: Writing—review & editing. AM: Writing—review & editing. GM: Writing—review & editing.
